# Plasma levels of Coenzyme Q10 are reduced in critically ill patients as compared to healthy volunteers and correlate with age

**DOI:** 10.1186/cc10761

**Published:** 2012-03-20

**Authors:** A Coppadoro, L Berra, A Kumar, M Yamada, R Pinciroli, E Bittner, U Schmidt, M Kaneki

**Affiliations:** 1 University of Milan-Bicocca, Monza, Italy; 2 Massachusetts General Hospital, Boston, MA, USA

## Introduction

The purpose of this study is to investigate Coenzyme Q10 (Q10) levels in critically ill patients as compared to healthy volunteers. Q10 is an essential cofactor for the electron transport chain reactions necessary for the aerobic cellular respiration. Q10 insufficiency, therefore, leads to mitochondrial dysfunction. It also acts as an antioxidant. Oxidative state is prominent in critically ill patients, favoring the production of oxygen-free radicals. A recent study showed reduced Q10 levels in septic shock patients [1].

## Methods

We recruited 18 healthy volunteers and 36 critically ill patients in the surgical ICU of the Massachusetts General Hospital. Ethical committee approval and written informed consent were obtained. At the moment of blood sampling, height, weight, and age as well as clinical data were collected. Plasma total Q10 concentrations were measured by high-performance liquid chromatography. The Assessment of Daily Living (ADL) score was obtained after discharge.

## Results

Patients' age and gender did not differ as compared to healthy volunteers (*P *= NS). Plasma Q10 levels were lower in critically ill patients as compared to healthy volunteers (0.81 ± 0.22 vs. 0.50 ± 0.36 μg/ml, *P *< 0.001). In critically ill patients, plasma Q10 levels inversely correlated with age (*R *= 0.40, *P *= 0.015). Lower levels of plasma Q10 (<0.4 μg/ml, median) were associated with lower ADL score after discharge (*P *= 0.005). In our patient population, plasma Q10 levels were not related to PaO_2_/FiO_2_, septic shock, SAPS 2 at ICU admission, SOFA score or mortality (all *P *= NS).

## Conclusion

Plasma Q10 levels are reduced in critically ill patients, suggesting reduced antioxidant capacity. Older patients seem to be more prone to exhibit low Q10 levels. Oral supplementation might be considered for those patients.
